# The genome sequence of the Panther Danio, *Danio aesculapii *Kullander & Fang, 2009

**DOI:** 10.12688/wellcomeopenres.25028.1

**Published:** 2025-10-10

**Authors:** Kerstin Howe, Braedan McCluskey, Zoltan Varga, Bill Trevarrow, Shane A. McCarthy, Sarah Pelan, Jonathan M. D. Wood, Michelle Smith, Karen Oliver

**Affiliations:** 1 Tree of Life, Wellcome Sanger Institute, Hinxton, England, UK; 2 Department of Biology, University of Virginia, Charlottesville, Virginia, USA; 3 Zebrafish International Resource Center (ZIRC), Eugene, Oregon, USA; 4 Eugene Research Aquatics LLC, University of Oregon, Eugene, Oregon, USA; 5 University of Cambridge Department of Genetics, Cambridge, England, UK; 6 Wellcome Sanger Institute, Hinxton, England, UK

**Keywords:** Danio aesculapii, Panther Danio, genome sequence, chromosomal, Cypriniformes

## Abstract

We present a genome assembly from a female specimen of *Danio aesculapii* (the Panther Danio; Chordata; Actinopteri; Cypriniformes; Cyprinidae). The genome sequence is 1,381.5 megabases in span. Most of the assembly is scaffolded into 25 chromosomal pseudomolecules. Gene annotation of this assembly on Ensembl identified 23,884 protein coding genes.

## Species taxonomy

Eukaryota; Metazoa; Eumetazoa; Bilateria; Deuterostomia; Chordata; Craniata; Vertebrata; Gnathostomata; Teleostomi; Euteleostomi; Actinopterygii; Actinopteri; Neopterygii; Teleostei; Osteoglossocephalai; Clupeocephala; Otomorpha; Ostariophysi; Otophysi; Cypriniphysae; Cypriniformes; Cyprinoidei; Danionidae; Danioninae; *Danio*; *Danio aesculapii* ( Kullander & Fang, 2009) (NCBI:txid1142201).

## Background

The Panther Danio ( *Danio aesculapii*) is a species within the Cyprinidae family, closely related to the well-studied zebrafish ( *Danio rerio*). Endemic to Myanmar, *D. aesculapii* is distinguished from other species in the Danio genus by its colour pattern comprising 6 to 7 brown vertical bars anteriorly on side and two horizontal rows of small brown spots posteriorly, as well as the absence of D stripe, and absence of dark stripes on caudal fin ( FishBase, 2024; Kullander & Fang, 2009).

This species has become a focal point in evolutionary biology and genomics due to its distinct morphological characteristics. The recent genomic research has elucidated some aspects of its genetic makeup and adaptive traits. Specifically, studies have highlighted the role of the potassium channel gene Kcnj13 in the diversification of its colour patterning ( Podobnik *et al.*, 2020). The comparative genomic analysis between *D. aesculapii* and *D. rerio* has provided valuable insights into the genetic basis of morphological variation and the evolutionary processes driving these differences ( McCluskey & Postlethwait, 2015).

In addition to its genetic studies, *Danio aesculapii* offers significant insights into the field of developmental biology, particularly in understanding the mechanisms of pigment cell interactions and pattern formation. The use of CRISPR/Cas9 gene-editing technology has been instrumental in dissecting the roles of specific genes in chromatophore interactions and patterning, shedding light on the evolutionary divergence in these processes compared to other *Danio* species.

As research continues, *Danio aesculapii* is expected to contribute significantly to our understanding of vertebrate development, genetics, and evolutionary biology, paralleling the contributions made by its more famous relative, the zebrafish.

## Genome sequence report

**Figure 1.**
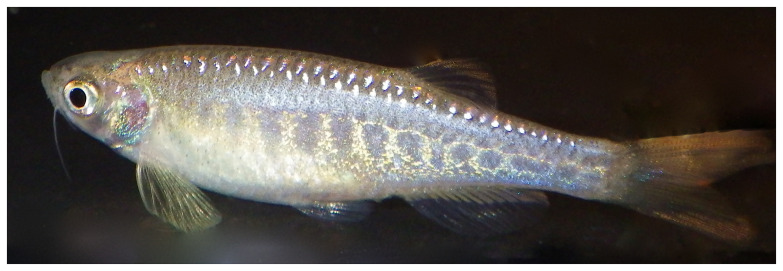
Image of *Danio aesculapii* (not the specimen used for genome sequencing). Photograph by Andrewbogott.

The genome was sequenced from a female *Danio aesculapii* ( Figure 1), based on a sample provided by Braedan McCluskey, UW at Seattle. A total of 41-fold coverage in Pacific Biosciences single-molecule CLR and 39-fold coverage in 10X Genomics read clouds was generated. Manual assembly curation corrected 460 missing joins or mis-joins and removed four haplotypic duplications. This reduced the total assembly length by 0.55%, the scaffold number by 35.35%, and increased the scaffold N50 by 74.95%.

**Figure 2.**
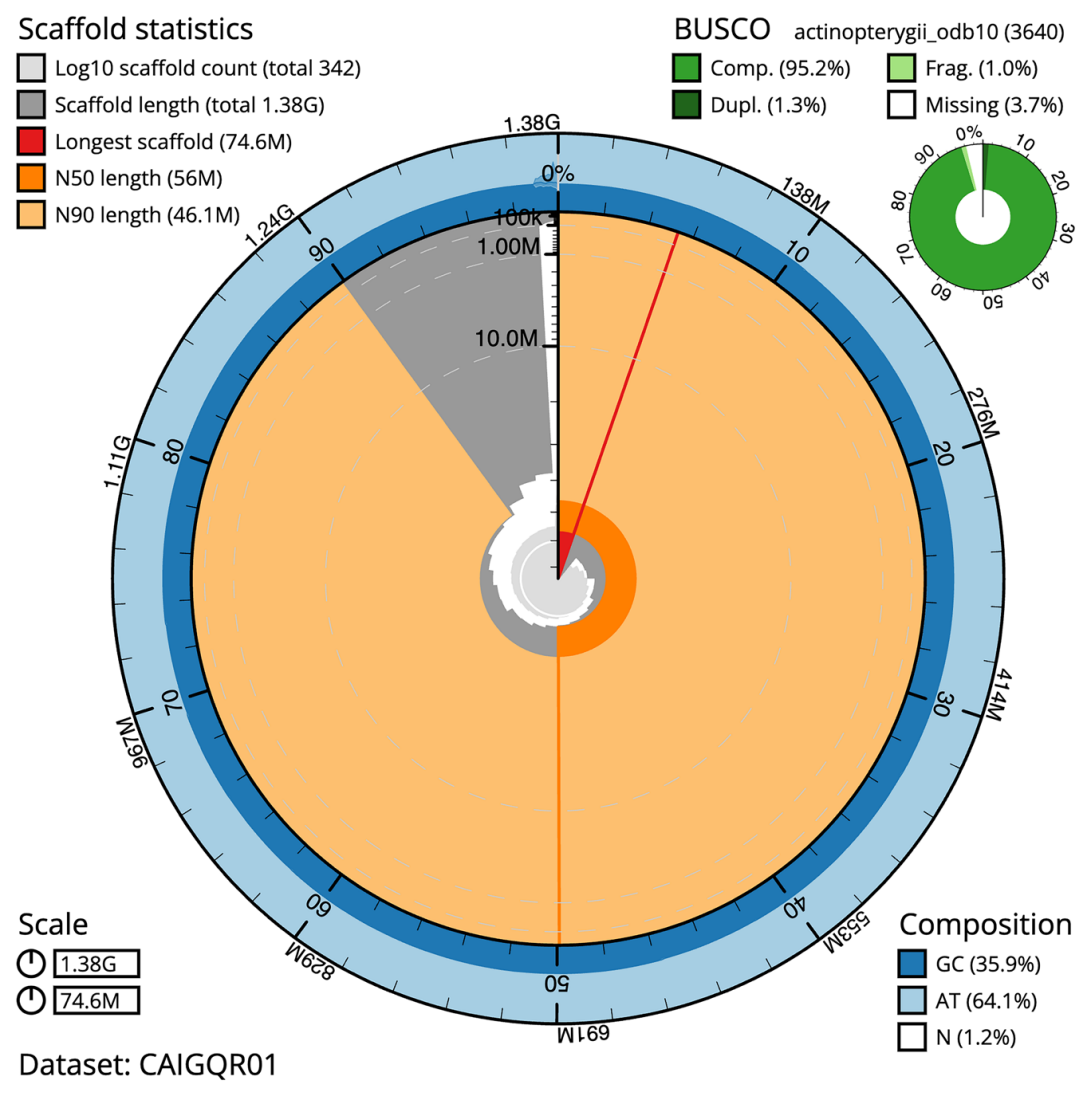
Genome assembly of *Danio aesculapii*, fDanAes4.1: metrics. The BlobToolKit Snailplot shows N50 metrics and BUSCO gene completeness. The main plot is divided into 1,000 size-ordered bins around the circumference with each bin representing 0.1% of the 1,381,485,597 bp assembly. The distribution of sequence lengths is shown in dark grey with the plot radius scaled to the longest sequence present in the assembly (74,595,647 bp, shown in red). Orange and pale-orange arcs show the N50 and N90 sequence lengths (55,996,547 and 46,123,639 bp), respectively. The pale grey spiral shows the cumulative sequence count on a log scale with white scale lines showing successive orders of magnitude. The blue and pale-blue area around the outside of the plot shows the distribution of GC, AT and N percentages in the same bins as the inner plot. A summary of complete, fragmented, duplicated and missing BUSCO genes in the actinopterygii_odb10 set is shown in the top right. An interactive version of this figure is available at https://blobtoolkit.genomehubs.org/view/GCA_903798145.1/dataset/CAIGQR01/snail.

**Figure 3.**
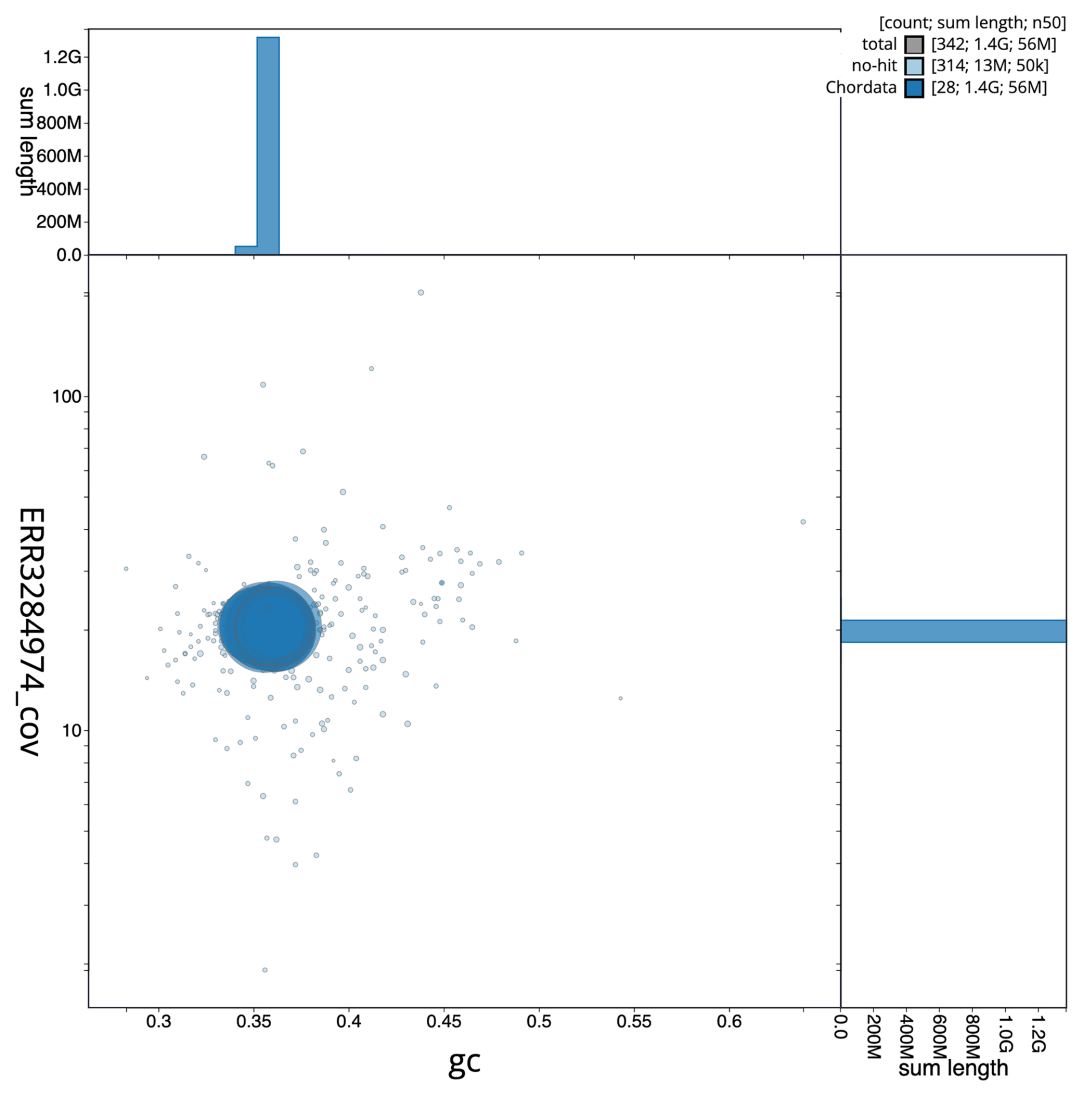
Genome assembly of *Danio aesculapii*, fDanAes4.1: BlobToolKit GC-coverage plot. Scaffolds are coloured by phylum. Circles are sized in proportion to scaffold length. Histograms show the distribution of scaffold length sum along each axis. An interactive version of this figure is available at https://blobtoolkit.genomehubs.org/view/GCA_903798145.1/dataset/CAIGQR01/blob.

**Figure 4.**
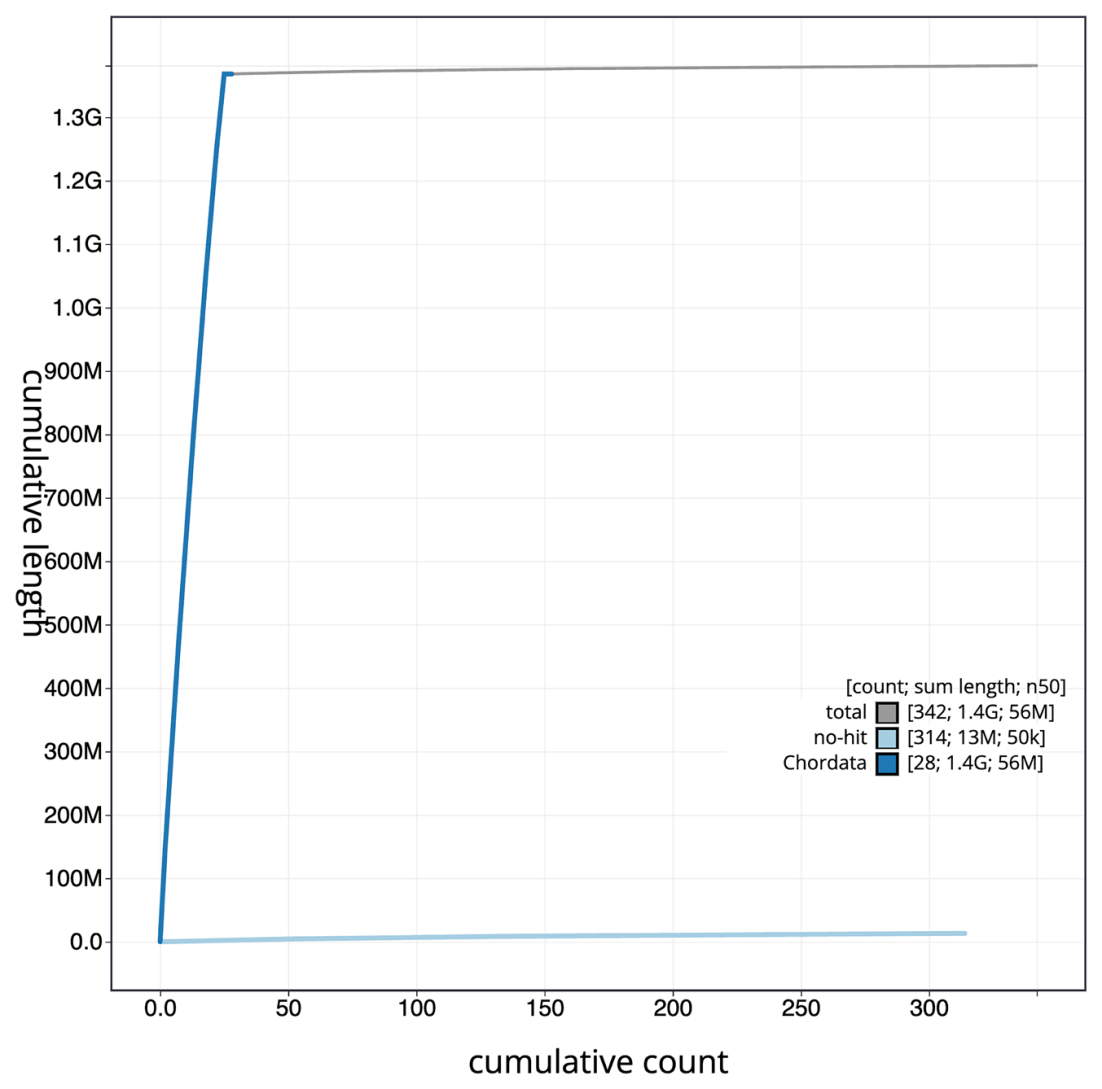
Genome assembly of *Danio aesculapii*, fDanAes4.1: BlobToolKit cumulative sequence plot. The grey line shows cumulative length for all scaffolds. Coloured lines show cumulative lengths of scaffolds assigned to each phylum using the buscogenes taxrule. An interactive version of this figure is available at https://blobtoolkit.genomehubs.org/view/GCA_903798145.1/dataset/CAIGQR01/cumulative.

**Figure 5.**
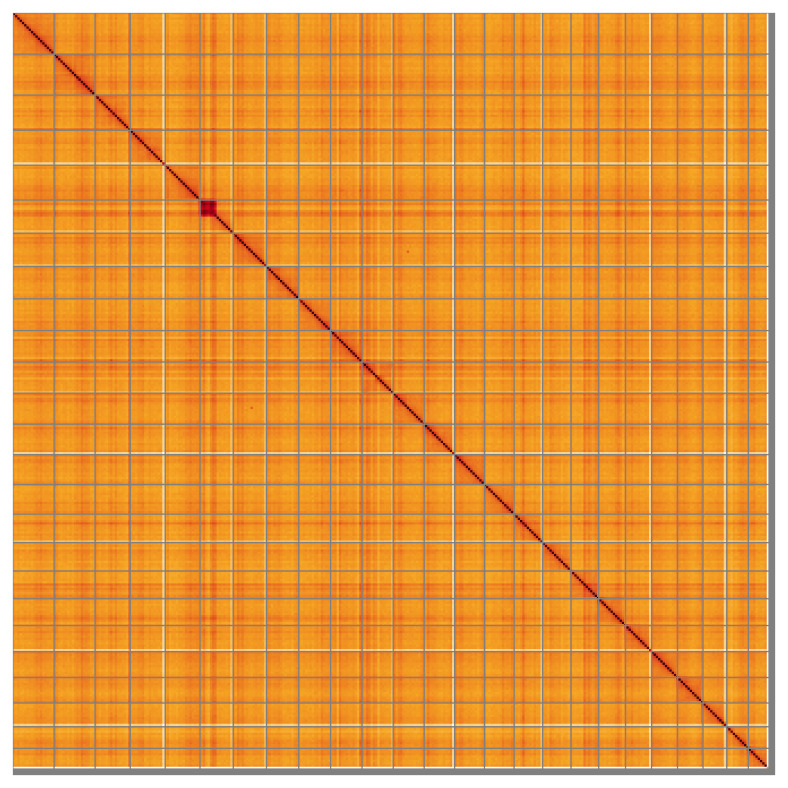
Genome assembly of *Danio aesculapii*, fDanAes4.1: Hi-C contact map of the fDanAes4.1 assembly, visualised using HiGlass. Chromosomes are shown in order of size from left to right and top to bottom. An interactive version of this figure may be viewed at https://genome-note-higlass.tol.sanger.ac.uk/l/?d=NqfPyxSZSEC7xRCzeZhxYA

The final assembly has a total length of 1,381.5 Mb in 342 sequence scaffolds with a scaffold N50 of 56.0 Mb ( Table 1). The snail plot in Figure 2 provides a summary of the assembly statistics, while the distribution of assembly scaffolds on GC proportion and coverage is shown in Figure 3. The cumulative assembly plot in Figure 4 shows curves for subsets of scaffolds assigned to different phyla. Most of the assembly sequence (99.03%) was assigned to 25 chromosomal-level scaffolds. Chromosome-scale scaffolds confirmed by the Hi-C data are named in order of size ( Figure 5; Table 2). While not fully phased, the assembly deposited is of one haplotype. Contigs corresponding to the second haplotype have also been deposited. The mitochondrial genome was also assembled and can be found as a contig within the multifasta file of the genome submission.

**Table 1.** Genome data for *Danio aesculapii*, fDanAes4.1.

Project accession data		
Assembly identifier	fDanAes4.1	
Species	*Danio aesculapii*	
Specimen	fDanAes4	
NCBI taxonomy ID	1142201	
BioProject	PRJEB38584	
BioSample ID	SAMEA104240212	
Isolate information	fDanAes4	
Assembly metrics	
Consensus quality (QV)	primary: 35.5; alternate: 32.9; combined: 34.1
*k*-mer completeness	primary:71.79%; alternate: 64.44%; combined: 93.13%
BUSCO *	C:95.2%[S:94.0%,D:1.3%], F:1.0%,M:3.7%,n:3,640
Percentage of assembly mapped to chromosomes	99.03%
Raw data accessions		
PacificBiosciences CLR	ERR3291642, ERR3291640, ERR3291641	
10X Genomics Illumina	ERR3332302, ERR3332303, ERR3332305, ERR3332304, ERR3284976, ERR3284973, ERR3284975, ERR3284974	
Hi-C Dovetail	ERR3655543	
Genome assembly		
Assembly accession	GCA_903798145.1	
*Accession of alternate haplotype*	GCA_903798135.1	
Span (Mb)	1,381.5	
Number of contigs	2,163	
Contig N50 length (Mb)	1.5	
Number of scaffolds	342	
Scaffold N50 length (Mb)	56.0	
Longest scaffold (Mb)	74.6	
Genome annotation		
Number of protein-coding genes	23,884	
Number of non-coding genes	2,236	
Number of gene transcripts	60,042	

* BUSCO scores based on the actinopterygii_odb10 BUSCO set using v5.3.2. C = complete [S = single copy, D = duplicated], F = fragmented, M = missing, n = number of orthologues in comparison. A full set of BUSCO scores is available at https://blobtoolkit.genomehubs.org/view/GCA_903798145.1/dataset/CAIGQR01/busco.

**Table 2.** Chromosomal pseudomolecules in the genome assembly of *Danio aesculapii*, fDanAes4.

INSDC accession	Chromosome	Length (Mb)	GC%
LR812505.1	1	63.56	36.0
LR812511.1	2	60.05	36.5
LR812517.1	3	62.92	36.5
LR812500.1	4	60.16	37.5
LR812498.1	5	74.6	36.0
LR812515.1	6	63.31	36.0
LR812512.1	7	74.25	36.5
LR812495.1	8	55.07	36.0
LR812510.1	9	58.43	36.0
LR812508.1	10	46.12	36.5
LR812506.1	11	47.05	36.0
LR812518.1	12	51.37	36.0
LR812503.1	13	53.68	36.0
LR812497.1	14	56.86	36.5
LR812516.1	15	48.49	36.5
LR812499.1	16	57.62	36.0
LR812496.1	17	53.95	36.0
LR812514.1	18	56.44	36.5
LR812507.1	19	51.62	36.0
LR812504.1	20	56.0	36.5
LR812501.1	21	46.98	36.0
LR812494.1	22	38.98	36.5
LR812513.1	23	50.12	36.5
LR812509.1	24	42.96	36.0
LR812502.1	25	37.52	36.0

The estimated Quality Value (QV) of the final assembly is 35.5 for the primary assembly, 32.9 for the alternate haplotype and 34.1 for the combined assemblies. The *k*-mer completeness was 71.79% for the primary, 64.44% for the alternate haplotype and 93.13% for the combined assemblies. The primary assembly has a BUSCO v5.3.2 completeness of 95.2% (single = 94.0%, duplicated = 1.3%), using the actinopterygii_odb10 reference set ( *n* = 3,640).

## Genome annotation report

The *Danio aesculapii* genome assembly (GCA_903798145.1) was annotated using the Ensembl rapid annotation pipeline ( Table 1; https://beta.ensembl.org/species/2f6daf5d-ae5d-4d81-ab77-65781ee1b41f). The resulting annotation includes 60,042 transcribed mRNAs from 23,884 protein-coding and 2,236 non-coding genes.

## Methods

### Sample acquisition and nucleic acid extraction

A female *Danio aesculapii* (sample accession SAMEA104240212, ToLID fDanAes4) was supplied by Braedan McCluskey.

Nucleic acid extraction was carried out using Bionano Prep Cell Culture DNA Isolation Protocol. In this method, the cells are first embedded in agarose to provide structural support during the extraction process. The agarose-embedded cells are then treated with lysis buffers to break down the cell membranes and release the DNA. The process also involves proteinase digestion to remove proteins, followed by a series of washes to purify the DNA.

### Sequencing

The assembly fDanAes4.1 is based on 52x CLR PacBio data, 71x 10X Genomics Chromium data, BioNano data and 32x Dovetail Hi-C data. Pacific Biosciences HiFi circular consensus and 10X Genomics read cloud DNA sequencing libraries were constructed according to the manufacturers’ instructions. DNA sequencing was performed by the Scientific Operations core at the WSI on Pacific Biosciences SEQUEL II (HiFi) and HiSeq X Ten (10X) instruments. Dovetail Hi-C data were also generated from tissue of fDanAes4 and sequenced on the HiSeq X Ten instrument.

### Genome assembly, curation and evaluation

The assembly fDanAes4.1 is based on 52x PacBio data, 71x 10X Genomics Chromium data, BioNano data and 32x Dovetail Hi-C data generated at the Wellcome Sanger Institute. The assembly process included the following sequence of steps: initial PacBio assembly generation with Falcon-unzip ( Chin *et al.*, 2016), retained haplotig separation with purge_dups ( Guan *et al.*, 2020), 10X based scaffolding with scaff10x, BioNano hybrid-scaffolding with Solve, Hi-C based scaffolding with SALSA2 ( Ghurye *et al.*, 2019), Arrow polishing, and two rounds of FreeBayes ( Garrison & Marth, 2012) polishing. Finally, the assembly was analysed and manually improved using the using gEVAL ( Chow *et al.*, 2016) as described previously ( Howe *et al.*, 2021). Chromosome-scale scaffolds are named by synteny to the GRCz11 assembly of *Danio rerio*.

A Hi-C map for the final assembly was produced using bwa-mem2 ( Vasimuddin *et al.*, 2019) in the Cooler file format ( Abdennur & Mirny, 2020). To assess the assembly metrics, the *k*-mer completeness and QV consensus quality values were calculated in Merqury ( Rhie *et al.*, 2020). The genome was analysed within the BlobToolKit environment ( Challis *et al.*, 2020) and BUSCO scores ( Manni *et al.*, 2021) were calculated.

Table 3 contains a list of relevant software tool versions and sources.

**Table 3.** Software tools: versions and sources.

Software tool	Version	Source
BlobToolKit	4.1.7	https://github.com/blobtoolkit/blobtoolkit
BUSCO	5.3.2	https://gitlab.com/ezlab/busco
FreeBayes	1.3.1-17-gaa2ace8	https://github.com/freebayes/freebayes
Falcon_unzip	0.4.0	https://github.com/PacificBiosciences/FALCON_unzip
gEVAL	N/A	https://geval.org.uk/
Hifiasm	0.12	https://github.com/chhylp123/hifiasm
HiGlass	1.11.6	https://github.com/higlass/higlass
Long Ranger ALIGN	2.2.2	https://support.10xgenomics.com/genome-exome/software/pipelines/latest/advanced/other-pipelines
Merqury.FK	1.1.2	https://github.com/thegenemyers/MERQURY.FK
MitoHiFi	2	https://github.com/marcelauliano/MitoHiFi
PretextView	0.2	https://github.com/wtsi-hpag/PretextView
purge_dups	1.2.3	https://github.com/dfguan/purge_dups
SALSA	2.2	https://github.com/salsa-rs/salsa

### Genome annotation

The Ensembl gene annotation system ( Aken *et al.*, 2016) was used to generate annotation for the *Danio aesculapii* assembly (GCA_903798145.1). Annotation was created primarily through alignment of transcriptomic data to the genome, with gap filling via protein-to-genome alignments of a select set of proteins from UniProt ( UniProt Consortium, 2019).

### Wellcome Sanger Institute – Legal and Governance

The materials that have contributed to this genome note have been supplied by a Darwin Tree of Life Partner. The submission of materials by a Darwin Tree of Life Partner is subject to the **‘Darwin Tree of Life Project Sampling Code of Practice’**, which can be found in full on the Darwin Tree of Life website here. By agreeing with and signing up to the Sampling Code of Practice, the Darwin Tree of Life Partner agrees they will meet the legal and ethical requirements and standards set out within this document in respect of all samples acquired for, and supplied to, the Darwin Tree of Life Project.

Further, the Wellcome Sanger Institute employs a process whereby due diligence is carried out proportionate to the nature of the materials themselves, and the circumstances under which they have been/are to be collected and provided for use. The purpose of this is to address and mitigate any potential legal and/or ethical implications of receipt and use of the materials as part of the research project, and to ensure that in doing so we align with best practice wherever possible. The overarching areas of consideration are:

•   Ethical review of provenance and sourcing of the material

•   Legality of collection, transfer and use (national and international)

Each transfer of samples is further undertaken according to a Research Collaboration Agreement or Material Transfer Agreement entered into by the Darwin Tree of Life Partner, Genome Research Limited (operating as the Wellcome Sanger Institute), and in some circumstances other Darwin Tree of Life collaborators.

## Data Availability

European Nucleotide Archive: *Danio aesculapii* (panther danio). Accession number PRJEB38584; https://identifiers.org/ena.embl/PRJEB38584. The genome sequence is released openly for reuse. The *Danio aesculapii* genome sequencing initiative is part of the Vertebrate Genomes Project project. All raw sequence data and the assembly have been deposited in INSDC databases. Raw data and assembly accession identifiers are reported in Table 1.
