# Supplementary material for: The genome sequence of the Panther Danio, Danio aesculapii Kullander & Fang, 2009
Source: Wellcome Open Res. 2025 Oct 10;10:560. [Version 1] doi: 10.12688/wellcomeopenres.25028.1 (PMC12669980; doi:10.12688/wellcomeopenres.25028.1)
Supplement: Peer review history

# Reviewer report: Reviewer response for version 1

**DOI:** 10.21956/wellcomeopenres.27586.r139056

**Published:** 2025-12-01

**Authors:** Karim Gharbi

**Affiliations:** 1 Earlham Institute, Norwich, England, UK

This data note reports on the sequencing, assembly and annotation of a chromosome-level reference for the Panther Danio, *Danio aesculapii*, a congeneric species of the zebrafish biomedical model. The authors provide a short introduction with basic information on the species and its use in research, followed by standard genome sequence and annotation reports, and methods.

 The introduction is informative and highlights the benefits of research carried out using the species alongside the more established *Danio rerio*. The genome sequence reports indicates that the authors have achieved chromosome-level contiguity. The annotation report is short, but metrics are generally consistent with the methods employed and findings in other teleost genomes. The methods section provides a good level of details but I have noted some inconsistencies and omissions: 

 1. Coverage figures for CLR PacBio and 10X Genomics read cloud data are different from those reported in the sequencing section of the genome sequence report. 

 2. The use of the term 'HiFi' for CLR PacBio reads is incorrect. These are distinct data types. 

 3. Sample and library preparation for Dovetail Hi-C data is missing details and a reference. 

 4. The Bionano Prep Cell Culture DNA Isolation Protocol is missing a reference. 

 Overall, the assembly represents a significant contribution to fish genomics and will more widely facilitate research in vertebrate developmental and evolutionary biology.

Are sufficient details of methods and materials provided to allow replication by others?

Partly

Is the rationale for creating the dataset(s) clearly described?

Yes

Are the datasets clearly presented in a useable and accessible format?

Partly

Are the protocols appropriate and is the work technically sound?

Yes

Reviewer Expertise:

Genomics, bioinformatics

I confirm that I have read this submission and believe that I have an appropriate level of expertise to confirm that it is of an acceptable scientific standard, however I have significant reservations, as outlined above.

# Reviewer report: Reviewer response for version 1

**DOI:** 10.21956/wellcomeopenres.27586.r139053

**Published:** 2025-12-01

**Authors:** Jayan Duminda M Senevirathna

**Affiliations:** 1 Uva Wellassa University, Badulla, Uva Province, Sri Lanka

The manuscript presents a well-executed and clearly written genome assembly for *Danio aesculapii*. The authors have produced a high-quality draft assembly supported by strong metrics, including a BUSCO completeness of 95%, which is fully acceptable for a vertebrate genome and indicates that the assembly captures the vast majority of expected conserved genes.

 The combination of adequate sequencing depth, appropriate assembly tools, and thorough contamination assessment (e.g., blob plot analysis) further supports the reliability of the assembly.

 The methods are described in sufficient detail to allow reproducibility, and the results are logically presented and well supported by figures and tables. The annotation workflow is appropriately selected, and the comparative genomic context enhances the biological relevance of the dataset. The manuscript is also strengthened by clear data accessibility statements.

 Overall, this is a solid and valuable contribution to fish genomics. Only minor improvements are suggested, such as adding clarification on annotation parameter settings and briefly discussing the small proportion of missing BUSCOs to provide context regarding potential assembly gaps or repetitive regions. With these minor revisions addressed, the manuscript is suitable for indexing.

Are sufficient details of methods and materials provided to allow replication by others?

Yes

Is the rationale for creating the dataset(s) clearly described?

Yes

Are the datasets clearly presented in a useable and accessible format?

Yes

Are the protocols appropriate and is the work technically sound?

Yes

Reviewer Expertise:

Aquatic Biotechnology and Genetics

I confirm that I have read this submission and believe that I have an appropriate level of expertise to confirm that it is of an acceptable scientific standard.

# Reviewer report: Reviewer response for version 1

**DOI:** 10.21956/wellcomeopenres.27586.r139049

**Published:** 2025-11-18

**Authors:** Bingpeng Xing

**Affiliations:** 1 Third Institute of Oceanography Ministry of Natural Resources, Xiamen, Fujian, China

1 The statement “A total of 41-fold coverage in Pacific Biosciences single-molecule CLR and 39-fold coverage in 10X Genomics read clouds was generated” is inconsistent with the later “Sequencing” section, which reports 52× CLR, 71× 10X, 32× Hi-C, and mentions that HiFi libraries were constructed. Please verify and report consistent coverage values throughout the manuscript. In addition, note that CLR and HiFi represent different sequencing technologies and should not be used interchangeably.

 2 The sentence “Most of the assembly sequence (99.03%) was assigned to 25 chromosomal-level scaffolds.” should be revised for precision: “In total, 99.03 % of sequences were assigned to 25 chromosomal pseudomolecules.”

 3 The sentence “The estimated Quality Value (QV) of the final assembly is 35.5 … The k-mer completeness was 71.79 % for the primary … and 93.13 % for the combined assemblies.” Please add a brief explanation clarifying that the relatively low primary completeness likely results from CLR read error rate, sample heterozygosity, and haplotype separation strategy. Indicate which reads (short-read or HiFi) were used as the k-mer database and the Merqury parameters applied. A one- or two-sentence note at the end of the Results or Methods section would suffice.

 4 Please provide the source, sequencing platform, data volume, and accession numbers for the transcriptomic data used in genome annotation.

 5 There is inconsistent use of Mb and Gb. The total genome size should be expressed uniformly in gigabases (Gb) throughout the text, tables, and figure legends.

Are sufficient details of methods and materials provided to allow replication by others?

Yes

Is the rationale for creating the dataset(s) clearly described?

Yes

Are the datasets clearly presented in a useable and accessible format?

Yes

Are the protocols appropriate and is the work technically sound?

Yes

Reviewer Expertise:

molecular biology， marine biodiversity，DNA，genome

I confirm that I have read this submission and believe that I have an appropriate level of expertise to confirm that it is of an acceptable scientific standard, however I have significant reservations, as outlined above.

# Reviewer report: Reviewer response for version 1

**DOI:** 10.21956/wellcomeopenres.27586.r136071

**Published:** 2025-10-14

**Authors:** Graham Etherington

**Affiliations:** 1 Earlham Institute, Norwich Research Park, Norwich, UK

The authors present a new assembly for *Danio aesculapii, *which is generated using a range of different genomics data and iteratively assembled into chromosomal pseudomolecules. 

 There is one issue with the paper's methods - under 'Genome Annotation' the authors state that annotation was created primarily through alignment of transcriptomic data to the genome, but there is no mention of this data in the sequencing report. They should state where this data comes from.

 Other minor comments include:

 Assembly size should be provided in Gbs, not Mbs.

 Remove the first sentence in the paragraph entitled 'Genome assembly, curation, and evaluation' - it's redundant, being written at the start the previous paragraph (add the Wellcome Sanger attribution to the previous paragraph).

 I'm unsure of why the authors ran BUSCO independently, as I thought the BlobToolkit does this (see Figure 2).

Are sufficient details of methods and materials provided to allow replication by others?

Yes

Is the rationale for creating the dataset(s) clearly described?

Yes

Are the datasets clearly presented in a useable and accessible format?

Partly

Are the protocols appropriate and is the work technically sound?

Yes

Reviewer Expertise:

Genome assembly, structural variant analyses, conservatino genomics.

I confirm that I have read this submission and believe that I have an appropriate level of expertise to confirm that it is of an acceptable scientific standard.
